# Gut microbiota composition in recurrent acute otitis media: a cross-sectional observational study

**DOI:** 10.1007/s12223-024-01174-z

**Published:** 2024-06-05

**Authors:** Andrej Florjan, Maja Rupnik, Aleksander Mahnic

**Affiliations:** 1 Department of Otorhinolaryngology and Cervicofacial Surgery, General Hospital Celje, Oblakova ulica 5, 3000 Celje, Slovenia; 2 Department for Microbiological Research, National Laboratory of Health, Environment and Food, Prvomajska ulica 1, 2000 Maribor, Slovenia; 3 Department of Microbiology, Faculty of Medicine, University of Maribor, Taborska ulica 8, 2000 Maribor, Slovenia

**Keywords:** Recurrent acute otitis media, Gut microbiota, Metagenomics, Nasopharynx, *Turicibacter*

## Abstract

Recurrent acute otitis media (rAOM) poses a significant challenge in children aged 1 to 6 years, characterized by frequent and treatment-resistant ear infections. While existing studies predominantly focus on alterations in the nasopharyngeal microbiome associated with rAOM, our research explores the understudied association with the gut microbiome. In this cross-sectional observational prospective study, we enrolled 35 children aged 1 to 6 years during the 2021/2022 cold season. The test group comprised children with rAOM (*n* = 16), and the control group consisted of generally healthy children (*n* = 19). Samples (stool and nasopharyngeal swabs) were collected in late spring to ensure an antibiotic-free period. Detailed metadata was gathered through a questionnaire examining factors potentially influencing microbiota. Microbiota composition was assessed through amplicon sequencing of the V3–V4 region of the 16S rRNA gene. Our findings revealed limited alterations in gut microbiota composition among children with rAOM compared to healthy controls. Six bacterial taxa (*Veillonella*, Lachnospiraceae, Ruminococcaceae, Lachnospiraceae, *Bacteroides* and *Blautia*) were differentially represented with weak statistical significance. However, several bacterial taxa displayed correlations with multiple consecutive infections, with *Turicibacter* showing the most significant association. Additionally, day care centre attendance emerged as a potent gut microbiota modifier, independent of rAOM. Although our study identified limited differences in gut microbiota composition between children with rAOM and healthy controls, the observed correlations between the number of infections and specific bacterial taxa suggest a potential link between rAOM and the gut microbiota, warranting further investigation.

**Supplementary Information:**

The online version contains supplementary material available at 10.1007/s12223-024-01174-z.

## Introduction

Recurrent otitis media (rAOM) is a prevalent condition characterized by repetitive middle ear infections, predominantly affecting young children aged between 6 months and 3 years, although it can also occur in older children. While uncomplicated otitis media is typically managed by paediatricians through medical history assessment and clinical examination, children with rAOM often require referral to otolaryngologists for further evaluation and potential surgical intervention (Goycoolea et al. 1991). The impact of rAOM extends beyond the affected individuals, imposing a significant socioeconomic burden on families. The consequences include healthcare costs, absenteeism, productivity loss, developmental delays in speech and language and reduced overall quality of life (Greenberg et al. 2003; Brouwer et al. 2005; Kujala et al. 2017).

Several well-known risk factors associated with rAOM include age (between 6 months and 2 years), genetic factors, passive smoking, allergies, craniofacial anomalies, immunodeficiency and snoring (Assiri et al. 2023). Implementing preventive measures, such as vaccinations, good hygiene practices and timely treatment of respiratory infections, can aid in reducing the risk and severity of rAOM (Dagan et al. 2016).

Probiotics have emerged as a potential intervention for rAOM, with some promising results reported in a review of 17 randomized controlled trials evaluating their efficacy in children with rAOM. However, caution is advised in interpreting these findings, as subgroup analyses highlighted the need for further research in this area (Scott et al. 2019). Dysbiosis, characterized by disruptions in gut microbiota composition and diversity, has been linked to immune dysregulation, potentially rendering individuals more susceptible to infections, including ear infections (Ihekweazu and Versalovic 2018; Willers and Viemann 2021; Zama et al. 2022).

In an observational study by Thapa et al. in 2020, the microbiota of children aged 1–6 undergoing classic otolaryngology operative procedures such as adenotonsillectomy and grommet insertion was analysed. They obtained nasopharyngeal and rectal swabs of children while under general anaesthesia. The study highlighted increase in *Haemophilus* spp. in nasopharyngeal microbiome of children who received antibiotics, but it lacked healthy controls (Thapa et al. 2020).

Only one study to date has investigated the gut microbiota in otitis media. The study analysed the MiBioGen consortium dataset, comparing 11 samples of acute suppurative otitis media and 16 samples of chronic suppurative otitis media against the remaining database using multiple logistic regression (Wang et al. 2023).

In our study, we aim to explore the potential association between gut microbiota alterations and rAOM in children. By investigating the composition and diversity of the gut microbiota in children with rAOM compared to healthy controls, we seek to deepen our understanding of different factors contributing to recurrent ear infections.

## Methods

### Sample collection

This cross-sectional observational prospective study included a total of 35 children aged between 1 and 6 years. The test group comprised children within the same age range who met the criteria for recurrent acute otitis media (rAOM). The control group consisted of healthy children without a history of otitis media within the last year and without associated chronic diseases. Recruitment of participants took place in collaboration with regional primary level paediatricians during the cold season of 2021/2022. For the test group, stool and nasopharyngeal swab samples were collected in late spring to ensure an antibiotic-free period. Control group stool samples were collected from healthy volunteers during routine check-ups. In total, 16 stool samples and 19 nasopharyngeal swabs were obtained from the test group, and 19 stool samples were collected from the control group. Additionally, detailed questionnaires were administered to the parents to gather metadata on factors that could potentially influence the microbiota composition (Supplementary file 1).

The study was approved by the ethical committee of Celje general hospital (27/KS/2022–1).

### Amplicon metagenomic sequencing

Total DNA was isolated from the collected samples using the QIAGEN mini kit. DNA concentrations were determined using the Quant-iT PicoGreen dsDNA Assay (Thermo Fisher Scientific, USA), and all samples were normalized to a concentration of 5 ng/µl. Libraries were prepared following the 16S Metagenomic Sequencing Library Preparation guide (Illumina, USA), targeting the V3–V4 hypervariable region of the 16S rRNA gene with broad-range primers 341F (5′–CCT ACG GGN GGC WGC AG–3′) and 805R (5′–GAC TAC HVG GGT ATC TAA TCC–3′) (Klindworth et al. 2013). Paired-end sequencing (2 × 300 bp) was performed on the MiSeq (Illumina, USA).

Data processing involved quality filtering of reads and construction of zero radius operational taxonomic units (ZOTUs) using the UNOISE3 pipeline implemented in USEARCH v.11.0.667 (Edgar 2010; Edgar et al. 2011) with default settings and the addition of -fastq-minlen 400 (fastq_filter command). Taxonomy was inferred using the RDP reference database v.18.

## Results and discussion

### Faecal and nasopharyngeal bacterial community characteristics

**Fig. 1:**
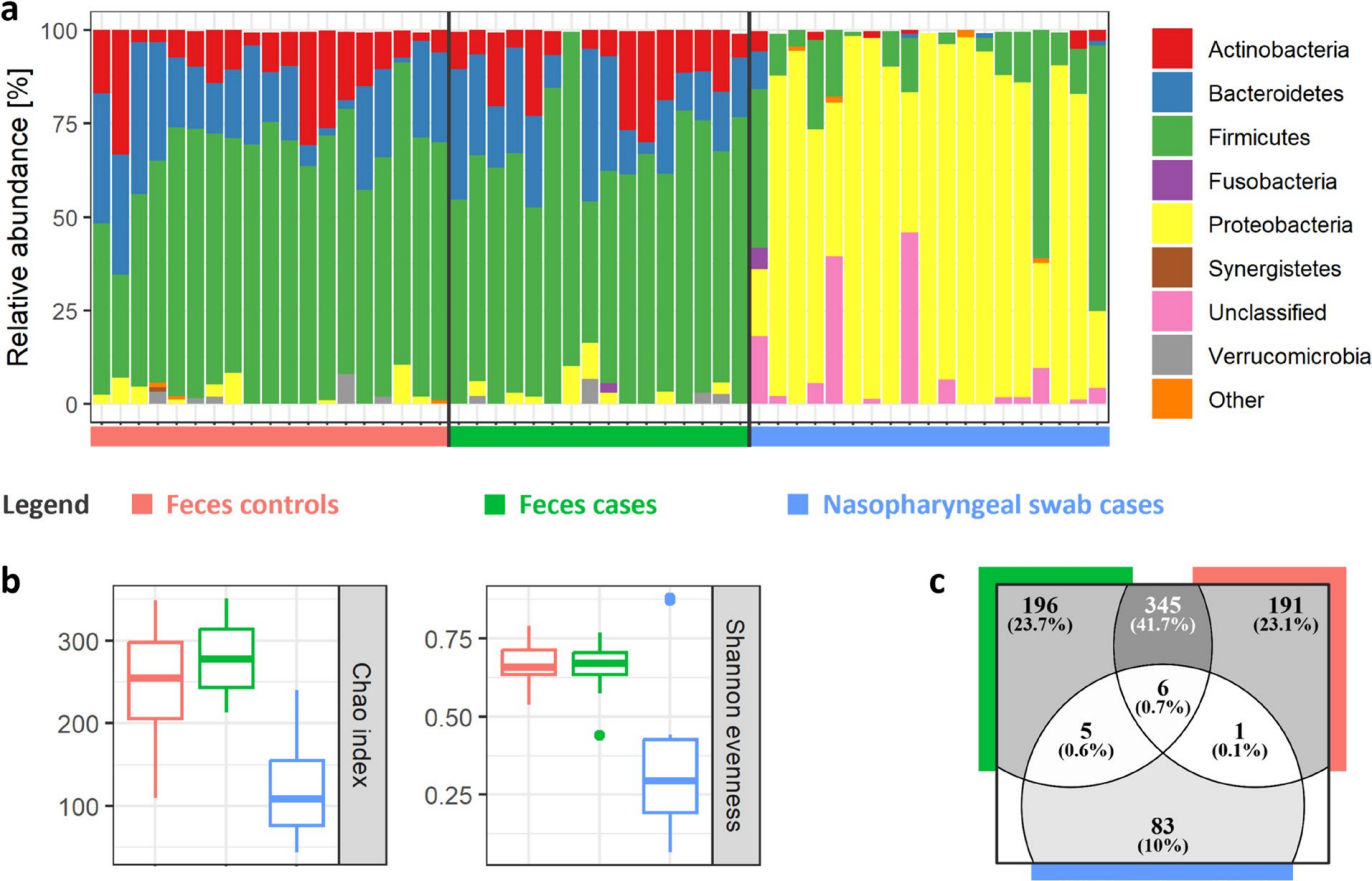
Faecal and nasopharyngeal bacterial community characteristics. The figure illustrates the characteristics of the faecal and nasopharyngeal bacterial communities. The data is presented separately for three compared groups: faecal microbiota in the test group (green), faecal microbiota in the control group (red) and nasopharyngeal microbiota in the test group (blue). **a** Relative abundance of bacterial phyla: this panel shows the relative abundance of bacterial phyla that are present at a relative abundance greater than 1% in each sample. **b** Alpha diversity analysis: this panel presents the results of the alpha diversity analysis, including the Chao index (a measure of community richness) and the Shannon evenness index (a measure of community evenness). **c** Venn diagram of shared ZOTUs: this diagram illustrates the overlap of ZOTUs among the three compared groups

Amplicon sequencing of the V3–V4 variable region of the 16S rRNA gene resulted in an average depth of 24,069.62 reads per sample, yielding a total of 905 distinct ZOTUs. The detected bacterial taxa encompassed 10 bacterial phyla. The faecal microbiota was predominantly composed of Firmicutes, Bacteroidetes and Actinobacteria. In contrast, the nasopharyngeal microbiota exhibited a dominance of Proteobacteria, particularly the genera *Moraxella* and *Haemophilus* (Fig. 1a). Certain nasopharyngeal swab samples exhibited a high relative abundance of unclassified taxa at the phylum level, highlighting a limited understanding and the lack of comprehensive databases pertaining to nasopharyngeal microbiota.

The richness of the nasopharyngeal microbiota was comparatively lower, with a median of approximately 100 ZOTUs per sample, contrasting with the > 250 ZOTUs observed in the faecal microbiota (Fig. 1b, Chao index). Furthermore, the nasopharyngeal microbiota exhibited significantly lower evenness compared to the faecal microbiota (Fig. 1b, Shannon evenness index). Specifically, the nasopharyngeal microbiota was frequently dominated by the genera *Moraxella*, *Haemophilus* and *Streptococcus*, which were also the most prevalent in the nasopharyngeal samples (prevalence 100.0%, 89.5% and 73.7% for *Moraxella, Haemophilus* and *Streptococcus*, respectively). This observation aligns with previous studies employing both metagenomic approaches (Nogues et al. 2020) and culture-based detection methods (Ngo et al. 2016).

In our study, the number of shared representatives between the faecal and nasopharyngeal microbiota was limited to 12 ZOTUs, and the number of shared representatives did not significantly differ between the test and control groups (Fisher exact test, *p* = 0.475; Fig. 1c). These included multiple representatives from genera Streptococcus (*n* = 6), *Veillonella* (*n* = 2), and a single representative from Corinebacteriaceae, *Haemophilus*, *Granulicatella* and *Gemella*. The mechanism of direct microbial translocation from the nasopharynx to the gut remains poorly understood, despite various established routes within the oral-gut axis, such as enteral, haematogenous and migration via immune cells (Tan et al. 2023). Although evidence regarding the successful colonization of the gut by oral microbiota is conflicting, heightened concentrations of oral microbes have been observed in several gastrointestinal disorders, including gastritis, inflammatory bowel disease, colorectal cancer and various chronic illnesses (Schmidt et al. 2019; Kitamoto et al. 2020). Recent investigations into the interplay between microbiota and COVID-19 have significantly advanced this field of research, notably by elucidating microbial networks between throat and gut microbiota (Xu et al. 2021). Studies have also highlighted disparities in the shared nasopharynx-gut microbiota between COVID-19 patients and healthy individuals (Mancabelli et al. 2023). However, it is worth noting that the study by Mancabelli et al. focused on adult patients and reported distinct shared microbiota patterns compared to our findings. Notably, no prior studies investigating nasopharynx-gut shared microbiota in paediatric populations were found.

### Gut microbiota composition in children with rAOM shows limited alteration compared to healthy controls

The gut microbiota community structure did not exhibit a significant difference between the test and control groups (PERMANOVA, *p* = 0.210; AMOVA, *p* = 0.842). Additionally, there was no discernible variation in richness and diversity between the compared groups of samples (community richness, *p* = 0.373; Shannon index, *p* = 0.894).

**Fig. 2:**
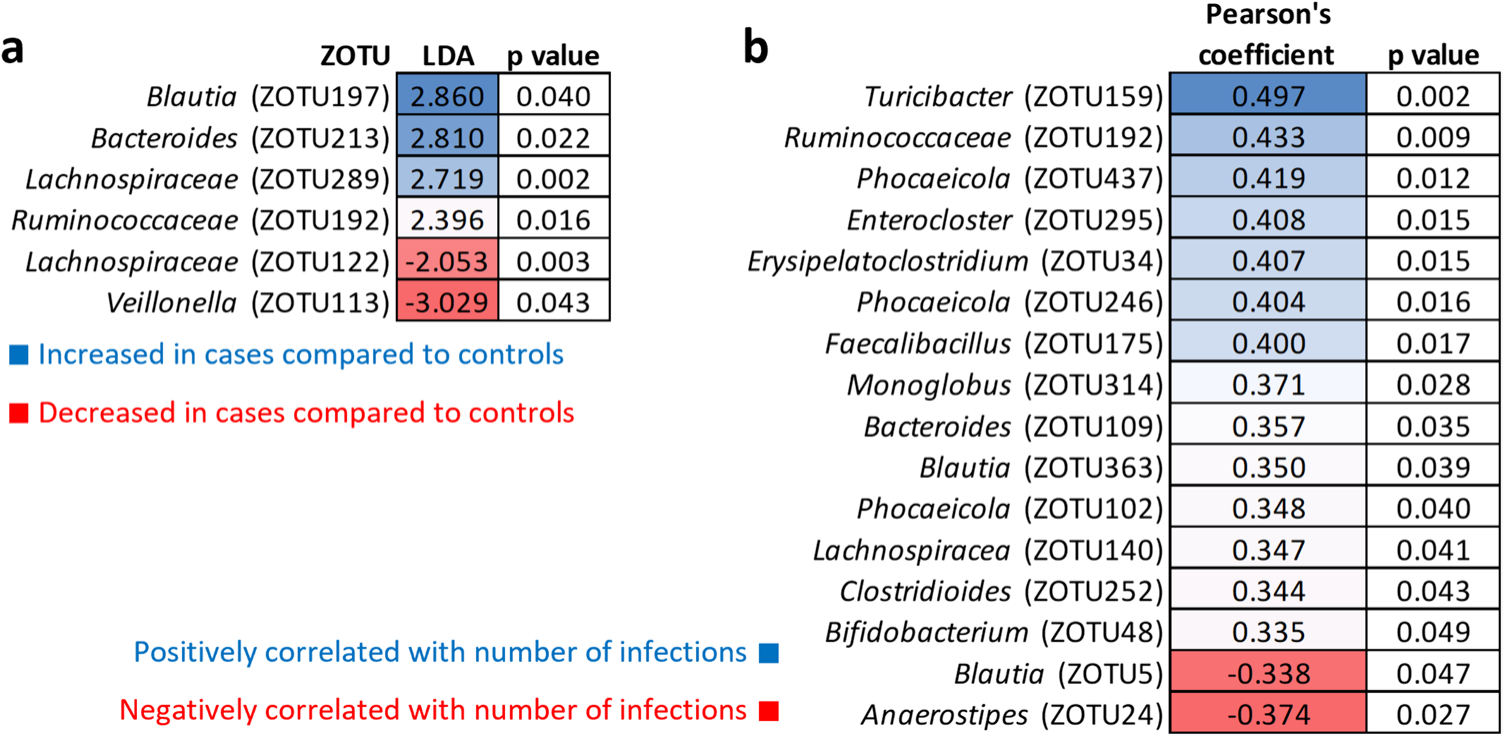
Gut microbiota characteristics of rAOM patients. The figure displays the results of **a** LEfSe test, highlighting the differentially abundant bacterial taxa between the test and control groups. The taxa that showed significant differences are represented by their corresponding linear discriminant analysis (LDA) scores. Negative LDA values (shown in red) indicate taxa that were decreased in the test group compared to the healthy controls, while positive LDA values (shown in blue) indicate taxa that were increased in the test group, and **b** Pearson’s correlation between bacterial taxa and number of infections

Upon conducting a population-level analysis, we identified six bacterial taxa that demonstrated weak statistical significance in their differential representation between the test and control groups. Specifically, *Veillonella* (ZOTU113) and Lachnospiraceae (ZOTU122) were decreased in the test group compared to the control group, whereas Ruminococcaceae (ZOTU192), Lachnospiraceae (ZOTU289), *Bacteroides* (ZOTU213) and *Blautia* (ZOTU197) were increased (Fig. 2a).

Wang et al. analysed the MiBioGen consortium dataset, comparing 11 samples of acute suppurative otitis media and 16 samples of chronic suppurative otitis media against the remaining database using multiple logistic regression. In MiBioGen consortium set, the authors identified a significant reduction in the Ruminococcaceae family in otitis media, which differs from our study’s findings, where increase was observed in case group and showed positive correlation to a number of infections (Fig. 2b). However, they did report a decrease in Lachnospiraceae in acute suppurative otitis media, which partially aligns with our observations (Vacca et al. 2020).

In our study, we observed weak correlations between the abundance of certain bacterial taxa and the number of infections recorded in the previous year (Fig. 2b). Among these correlations, the most significant ones were positive associations with *Turicibacter* (ZOTU159) and Ruminococcaceae (ZOTU192).

Ruminococcaceae is a diverse family of commensal bacteria, and further investigation is required to discuss the biological significance of the observed increase in relative abundance in our dataset. *Turicibacter* is a member of the commensal gut microbiota; however, previous studies have reported its increased abundance in *Helicobacter pylori* infection (Kienesberger et al. 2016) and rheumatoid arthritis (Chen et al. 2016). It has also been found to be increased in mice with depleted CD8 + T cells (Presley et al. 2010) and isolated from the blood culture of an acute appendicitis patient (Bosshard et al. 2002). Nonetheless, the underlying mechanisms of involvement of *Turicibacter* in immune responses have not yet been discovered. It is important to note that while these associations have been observed, the specific role of *Turicibacter* in immune response and its implications in infection remain to be elucidated.

### Host-associated features show limited correlation with faecal bacterial community structure

In total, we tested 16 features for their association with the composition of the faecal microbiota. Among these, we found that visiting kindergarten, as opposed to staying at home, was the only feature significantly associated with the bacterial community, explaining 6.9% of the interindividual variation (permutational multivariate analysis of variance (PERMANOVA) using Bray–Curtis distances, *p* = 0.006; Table 1). Visiting day care facilities was primarily linked to higher bacterial diversity compared to home care, as indicated by the (Shannon index, *p* = 0.022).

**Table 1:** Cohort characteristics and association with bacterial community structure based on permutational multivariate analysis of variance (PERMANOVA)

**Feature**	**Cases**	**Controls**	***p*** **value**
Group	*n* = 16	*n* = 19	0.194
Gender [male/female]	10/6	10/9	0.474
Age [mean ± SD]	1.75 ± 0.86	1.63 ± 0.83	0.199
Number of antibiotic therapies in previous year [mean ± SD]	5.50 ± 1.97	0.21 ± 0.54	0.813
Diabetes during pregnancy [yes/no]	1/15	3/16	0.305
Gestational age at delivery [mean ± SD]	39.50 ± 1.75	38.76 ± 1.39	0.059
Caesarean delivery [yes/no]	7/9	4/15	0.669
Birth weight in grams [mean ± SD]	3596.25 ± 462.09	3371.33 ± 563.72	0.223
Months exclusively breastfeeding [mean ± SD]	10.69 ± 6.50	10.53 ± 4.80	0.070
Probiotics [no/during antibiotic therapy/occasionally/regularly]	3/5/5/3	6/0/12/1	0.692
Day care facilities/home care	15/1	16/3	**0.033**
Family with more than one child [yes/no]	13/3	8/11	0.149
Exposure to domestic animals [yes/no]	10/6	11/8	0.270
Number of ear infections in previous year [mean ± SD]	5.56 ± 1.96	0.11 ± 0.46	0.819
Open mouth breathing [yes/no]	4/12	2/17	0.537
Removal of the oropharynx/tonsils [yes/no]	2/14	0/19	0.743

Statistically significant environmental variable is highlighted in bold

Interestingly, there is a scarcity of studies investigating differences in gut microbiota between children in day care facilities and home care. The only comprehensive study with well-defined groups of children reported findings which agree with our results (Amir et al. 2022). Specifically, children in day care facilities exhibited higher microbiota diversity. Additionally, they found that gut microbiota composition of day care children was more similar to that of adults compared to children in home care (Amir et al. 2022). In contrast, study by Gerben et al. reported no significant differences between children in day care and home care (Hermes et al. 2020). However, it is important to note that in this study, the follow-up period after children entered day care was only 4 weeks. This short duration is likely insufficient to observe significant changes in the gut microbiota.

We acknowledge that the low sample size was a major limitation of this study, particularly given the rapid changes in the microbiota of children under 4 years of age and the potential impact of external factors such as antibiotic therapy on community characteristics.

In conclusion, our study found limited differences in gut microbiota composition between children with rAOM and healthy controls. Interestingly, we observed a correlation between experiencing multiple consecutive infections and a higher relative abundance of *Turicibacter* in the gut. This genus has previously been reported to increase in various infection-associated conditions. Furthermore, regardless of health status, our findings confirmed previous research indicating that children in day care have higher gut microbiota diversity compared to those in home care.

## Supplementary Information

Below is the link to the electronic supplementary material.

Supplementary file1 (XLSX 14 KB)

## Data Availability

Amplicon sequencing data is available in NCBI SRA archive under BioProject accession number PRJNA1014361.
